# Influence of pain location and hand dominance on scapular kinematics and EMG activities: an exploratory study

**DOI:** 10.1186/1471-2474-12-267

**Published:** 2011-11-24

**Authors:** Yi-Fen Shih, Yi-Hsuan Kao

**Affiliations:** 1 Department of Physical Therapy and Assistive Technology, National Yang-Ming University, Taipei, Taiwan 112

## Abstract

**Background:**

Assessment of three-dimensional kinematics and electromyography (EMG) activities is common in patients with chronic neck pain. However, the effect of hand dominance and neck pain location on the measurement of movement and EMG characteristics is still unclear. Therefore, the purpose of this study was to investigate the effect of neck pain location and arm dominance on the scapular kinematics and muscle EMG activities in patients with chronic neck pain.

**Methods:**

Thirty subjects (10 males, 20 females; mean age (sd): 38 (11.9) years) with chronic neck pain for more than 3 months were recruited. The scapular kinematics and EMG activity of the upper trapezius and sternocleidomastoid muscles were measured during the bilateral arm elevation task. The three-way repeated measures ANOVA was used to examine the effect of neck pain location and hand dominance on the measurement of kinematics and EMG muscle activities.

**Results:**

The movement of scapular posterior tilt was significantly influenced by arm dominance (P = 0.001) and by the interaction of arm dominance and elevation angle (P = 0.002). The movement of scapular upward/downward rotation was affected by the interaction of arm dominance and elevation angle (P = 0.02). The location of pain did not show any significant influence on the scapular movement and muscle activities.

**Conclusions:**

Hand dominance could have an influence on the scapular kinematics, which should be taken into consideration when describing and comparing neuromuscular characteristics in individuals with chronic neck pain.

## Background

Measurement of three-dimensional kinematics and electromyography (EMG) activities is a common method to describe movement pattern and muscle performance in patients with chronic neck pain. Although most of the previous studies identified altered upper trunk postural control and muscle activation patterns during various testing tasks in subjects with chronic neck discomfort, none of these studies took into account the interaction of neck pain location and hand dominance on the neuromuscular performance in patients with chronic neck problems [1-5].

In healthy subjects, the effect of hand dominance on the neuromuscular control characteristics was examined by several researchers. Findings of Freitas et al. (2009), Crosbie et al. (2008) and Matsuki et al. (2011) supported the influence of hand dominance on movement patterns of the upper extremity and shoulder complex during dynamic activities [6,7]. Crosbie et al. (2008) compared the scapular kinematics between dominant and non-dominant arms and suggested that the side-differences in scapular kinematics during arm elevation tasks might relate to the difference in scapular muscle strength or neuromuscular control between sides [7]. Freitas et al. (2009) examined hemispheric differences in the variability of joint configurations during upper extremity reaching tasks and found bilateral variance in motor planning and execution [6]. A recent study by Matsuki et al. (2011) also identified increased dominant scapular upward and downward rotation at rest and during arm elevation [8]. The authors attributed these differences to bilateral variations in soft tissue balance, and suggested that these differences should be considered in clinical assessment [8]. Bagesteiro et al. (2002), Yoshizaki et al. (2009), and Diederichsen et al. (2007) nevertheless observed a similar kinematic pattern but a different EMG profile between the dominant and non-dominant arm [9-11]. Although the influence of hand dominance on the movement and muscle activities of the shoulder complex was evident in healthy subjects, no previous study investigated the effect of handedness in subjects with chronic neck pain.

Numerous studies demonstrated an altered movement control strategy and muscle activation pattern of the neck and scapular region in individuals with chronic neck disorders [1-5]. These studies used either functional tasks or arm elevation to assess the neuromuscular performance of the subjects. However, the possible influence of hand dominance and the location of pain had never been mentioned in these investigations. In the study by Lee and associates (2005), location of neck pain to one side was found to relate to decreased neck range of motion, and the amount of reduction in the neck rotation range was significantly greater on the side opposite to the pain. Since the location of pain, i.e. pain to dominant or non-dominant side, or pain on both sides, had a significant impact on the neck range of motion performance, this factor might also play a role in kinematics and muscle activities of the neck and shoulder complex in individuals with neck pain [12].

In summary, despite the evidence supporting the influence of handedness on scapular movement and muscle activity as well as the effect of pain location on the neck range of movement, published kinematic data in patients with chronic neck problems did not take into account these factors. Therefore, we designed this study to investigate the effect of neck pain location and hand dominance on the scapular kinematics and muscle EMG activities in patients with chronic neck pain. The hypothesis of the study was that movement of the scapula and the activity of the sternocleidomastoid and upper trapezius muscles would differ between the dominant and non-dominant arm, and between individuals with different neck pain location (neck pain on the side of dominant arm, non-dominant arm, or on both sides).

## Methods

### Subjects and inclusion/exclusion criteria

Thirty subjects with chronic neck pain for more than 3 months were recruited. Subjective information regarding patients' neck pain history, activity level, as well as their employment status was collected. Symptoms of these subjects had to be muscle-related or posture-related, and the area of symptoms had to be at the back of the neck or around the upper back/bilateral scapular region. We excluded patients whose subjective complaints indicated any neurological signs such as tingling or numbness. Assessment of the neck and shoulder region was conducted to rule out possible neurological conditions or joint disorders. Subjects with the following conditions were also excluded from the study: 1) trauma or surgery history of cervical spine; 2) neurological signs or symptoms, ex: motor weakness, numbness; 3) vestibular impairment, ex: dizziness, vertigo, motor imbalance; 4) malignancy; 5) other musculoskeletal disorders that might affect the completion of testing tasks; 6) participation in therapeutic exercise programs in the past 3 months. The location of neck pain, arm dominance, age, height, weight and Neck Disability Index (NDI) were recorded. The subjects were allocated into 3 groups based on the location of neck pain: pain on the dominant side, pain on the non-dominant side, and pain on both sides. All the subjects were informed of the purposes and procedures of this study and signed the consent form that was approved by the Institutional Review Board of National Yang Ming University, Taipei, Taiwan (serial number: 970027R). The protocol of this project conformed to the WMA Declaration of Helsinki-Ethical Principles for Medical Research Involving Human Subjects, and to the local legislation.

### Measurements

We used a three-dimensional electromagnetic tracking system (Liberty Polhemus, Colchester, VT, USA) to collect the three-dimensional kinematic data of scapula at 120 Hz sampling rate. Electromagnetic sensors were placed at the 3^rd ^thoracic spinal process, the flat bony surfaces of bilateral posterior-lateral acromions, and the posterior aspect of distal humerus on both arms [13]. A set of bony landmarks were manually palpated, labelled, and digitized based on the recommendations of the International Society of Biomechanics (ISB), to define the segmental coordinate systems [14].

An 8-channel FM/FM Telemetric EMG system (Telemyo 900, Noraxon USA, Inc., AZ, USA) was used to capture the electromyographic (EMG) activities of bilateral upper trapezius (UT) and sternal head of sternocleidomastoid (SCM) muscles at the sampling rate of 1000 Hz. We chose to observe UT and SCM muscles as abnormal firing patterns of these two muscles during arm elevation task have been observed in patients with chronic neck pain [2,3,15-18]. The EMG signals were sent through an A/D board (USB-1616HS-4, Measurement Computing, Norton, MA, USA) and recorded simultaneously with the kinematic data using the Motion Monitor^© ^software. The surface EMG electrodes used in this study were disposable bipolar silver/silver chloride electrodes (Blue Sensor P-00-S, Ambu Inc., Linthicum, USA) with a 2-cm inter-electrode distance. To reduce the skin impedance, the skin was shaved if needed, and cleaned and swabbed with cotton balls and 75% alcohol solution before electrode placement. The electrodes were positioned along the muscle fibers of UT at the midpoint of 7^th ^spinal process of cervical spine and acromion [2]. For the sternal head of SCM muscle, electrodes were placed along the muscle fiber at the one third point of the line from the sternal notch to the mastoid process [18]. The ground electrode was fixed at the right clavicle.

**Figure 1:**
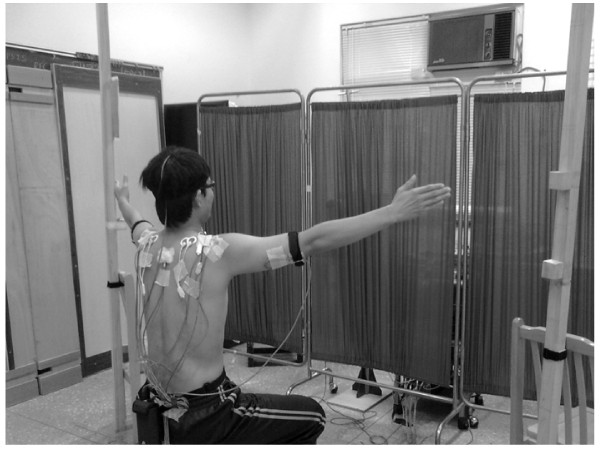
**Illustration of the experimental set-up**.

The testing task was a set of 3 repetitions of arm elevation in the scapular plane (about 30° anterior to the frontal plane). We marked the scapular plane on a wooden frame to guide the testing movement (Figure 1). The subject was encouraged to elevate their arms as high as possible in the sitting position. The speed of arm movement was guided by a metronome, with 4-second arm ascending and 4-second arm descending. The participants of our pilot study found this speed of movement easy to perform and did not cause any pain or discomfort during the movement. Before the data collection, subjects were asked to practice several times to familiarize with the speed and movement pattern of the testing task.

The digitization information and the kinematic data were calculated using the MotionMonitor^© ^software (Innovative Sports Training, Inc., Chicago, USA). The rotation center of the humerus was estimated by a least squares algorithm and defined as the point that moved the least during a sequence of passive shoulder rotation [19,20]. Standard matrix transformation with the Eular angle sequences were used to calculate the rotational matrix of bilateral scapulae and humerus in respect to the thorax. The humeral rotation sequence was firstly Yh (humerus horizontal adduction), secondly Xh (humerus elevation) and lastly Yh (+/-: humerus internal/external rotation). The rotation sequence of scapulae was Ys (+/-: scapula internal/external rotation), Xs (+/-: scapula downward/upward rotation) then finally Zs (+/-: scapula posterior/anterior tilt). The data was exported as ASCII files, and sorted using a customized Labview program (National Instruments, Inc, Austin, TX, USA). The kinematic data of bilateral scapulae during arm elevation were interpolated at 30°, 60°, 90° and 120° of humeral elevation. As we found highly consistent scapular kinematics across 3 trials of testing, data of the three trials were averaged for further analysis.

The raw EMG signals of the upper trapezius and sternocleidomastoid muscles were filtered by a customized Matlab program (The Mathworks Inc., Natick, MA) to eliminate the electrocardiogram signals, and then were band-pass filtered between 20 and 500 Hz and band-stop filtered between 58-62 Hz (Butterworth). For the EMG signals collected during bilateral arm elevation, the data were extracted according to the humeral elevation angle. Root-mean square (RMS) values of EMG data were averaged for the window of 30°-60°, 60°-90°, 90°-120°, 120°-90°, 90°-60°, and 60°-30°of the humeral elevation. The peak ensemble average normalization method was used to normalize the EMG data during the task [21-23].

The accuracy of the motion capture system and reliability of the kinematic and EMG measurements were examined in our pilot study. The set-up of the motion capture system had an accuracy of 0.3 to 0.7 degrees for the rotational movement and 0.4 cm for the translational movement. The intra-rater between session measurement repeatability, represented by intraclass correlation coefficient (ICC_3,3_), for the scapular kinematics was between 0.68 and 0.91, except 0.56 for scapular posterior tilt at 30 degrees of arm descending. The ICC values for the EMG measurement of SCM and UT muscles were generally between 0.70 and 0.94, except 0.55 for left UT muscle during 90-60 degrees of arm descending. The SEM values ranged from 0.85 to 4.22 degrees for the measurement of scapular kinematics and from 1.55% to 9.29% for the EMG measurement SCM and UT muscles.

### Statistical analysis

Chi-square test and one-way ANOVA were used to compare the basic data of the three groups. One-way ANOVA was also used to confirm the consistency of the scapular kinematics across three trials of testing. Three-way repeated measures ANOVA was used to examine the effect of neck pain location and arm dominance on kinematics and EMG muscle activities during bilateral arm elevation. The independent variables were neck pain location (neck pain on the side of the dominant arm, neck pain on the side of the non-dominant arm, neck pain on both sides), hand dominance (dominant and non-dominant arm), and angle of arm elevation (30°, 60°, 90° and 120°). The independent t-test was used as post-hoc tests if any significant interaction or main effect was identified. The α level was set at 0.05 for statistical significance. The significance level for the post-hoc tests were adjusted to 0.01 for multiple comparisons. All statistical analyses were performed with the Statistical Package for Social Sciences version 15.0 (SPSS 15.0, SPSS Inc., Chicago, USA).

## Results

### Subject description

Of the 30 subjects with chronic neck pain (10 males, 20 females; mean age (sd): 38 (11.9) years), nine had neck pain on the dominant side, nine had neck pain on the non-dominant side, and the other twelve subjects had pain on both sides of the neck and upper back area. Among the subjects, there were 12 students or research assistants, 6 desk workers, 5 housekeepers, 3 teachers, 2 consultants, 1 physical therapist, and 1 janitor. None of the subjects in this study reported neck pain or discomfort during the bilateral arm elevation testing task. The basic data of the three groups are summarized in Table 1. There was no significant difference in the basic data between the three groups.

**Table 1:** Comparisons of the basic data of the three study groups: mean (sd)

	Pain on dominant side (n = 9)	Pain on non-dominant side (n = 9)	Pain on both sides (n = 12)	P value
Gender (M:F)	1: 8	5:4	4:8	0.14
Height (cm)	158.75 (7.34)	165.33 (10.91)	162.50 (7.72)	0.26
Weight (kg)	56.50 (8.72)	61.56 (10.98)	61.30 (11.28)	0.55
Age (yrs)	35.3 (13.3)	41.4 (10.9)	37.3 (13.2)	0.71
Neck Disability Index	11.9 (2.16)	13.6 (6.0)	14.3 (5.1)	0.64

### Scapular kinematics

**Figure 2:**
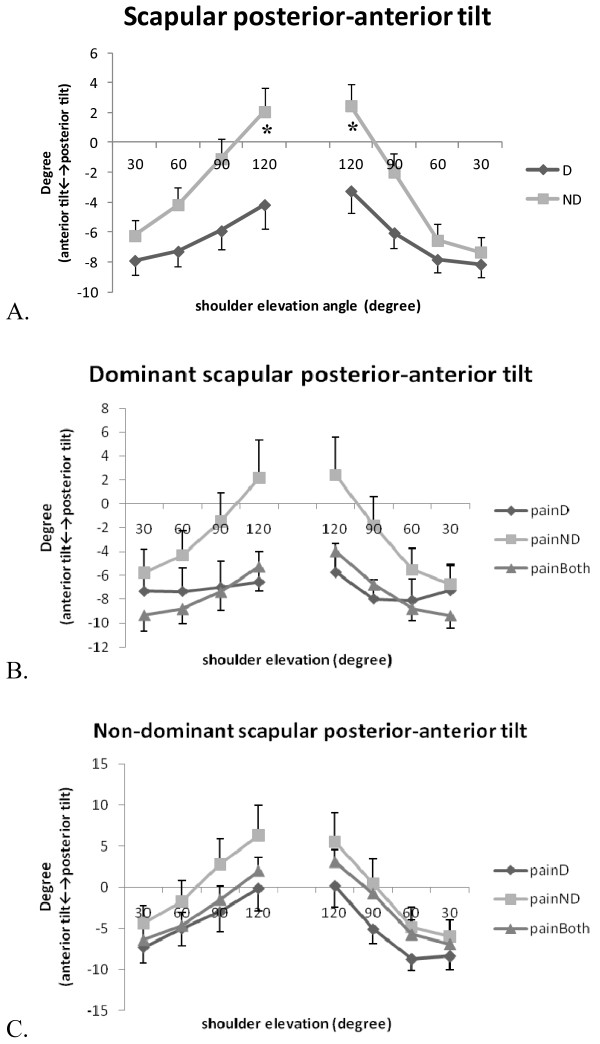
**Scapular posterior/anterior tilt during bilateral arm elevation**. (A) Comparisons between the dominant and non-dominant scapulae; (B) Comparisons of the three groups of the dominant scapula; (C) Comparisons of the three groups of the non-dominant scapula. D: dominant; ND: non-dominant; painD:pain on dominant side; painND: pain on non-dominant side; painBoth: pain on both sides. *: significant difference between dominant and non-dominant scapulae, P < 0.01.

**Figure 3:**
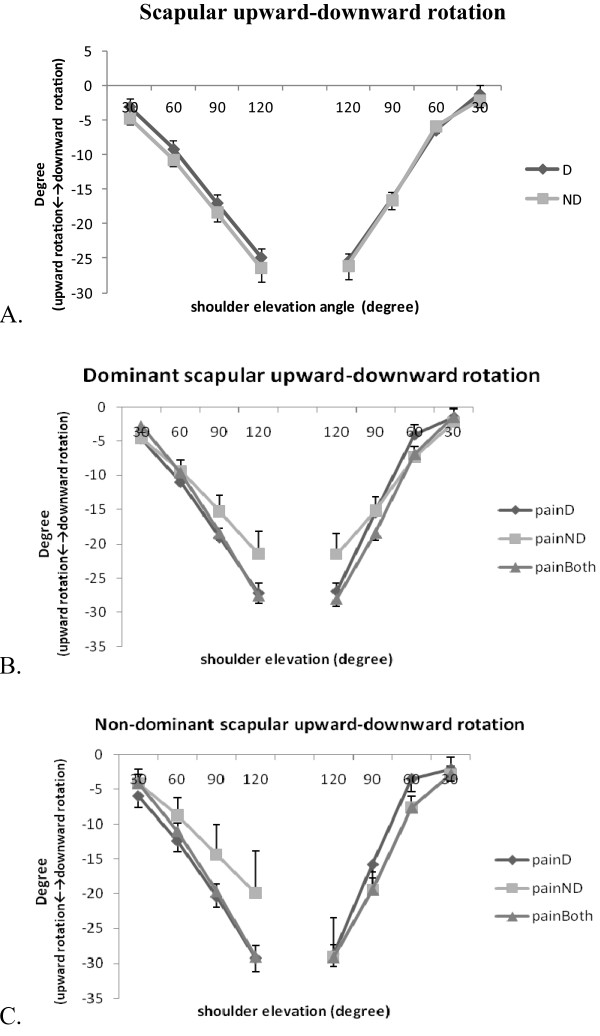
**Scapular upward/downward rotation during bilateral arm elevation**. (A) Comparisons between the dominant and non-dominant scapulae; (B) Comparisons of the three groups of the dominant scapula; (C) Comparisons of the three groups of the non-dominant scapula. D: dominant; ND: non-dominant; painD:pain on dominant side; painND: pain on non-dominant side; painBoth: pain on both sides.

**Figure 4:**
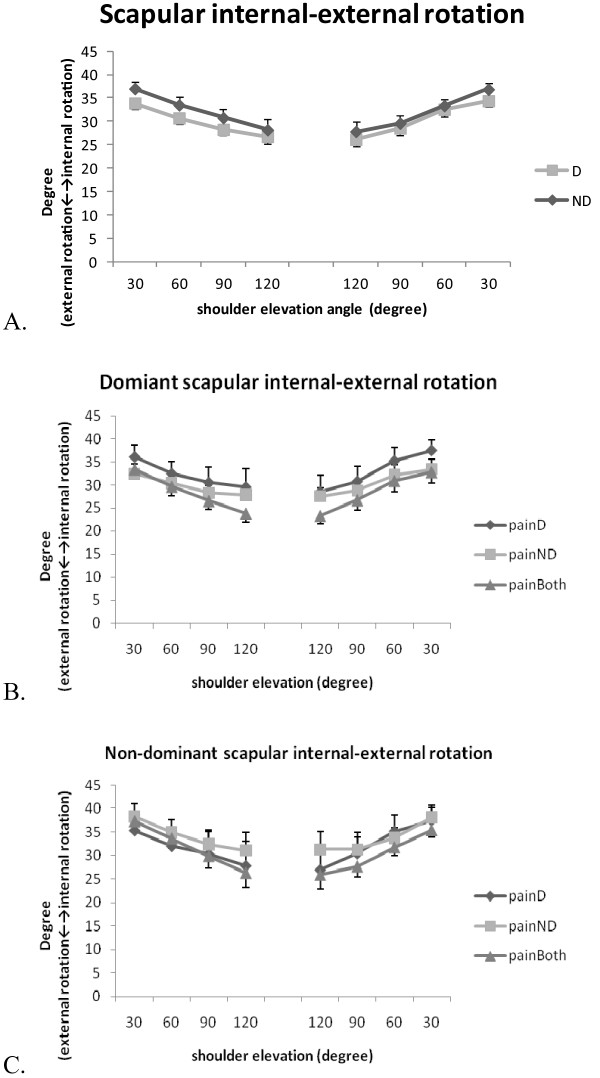
**Scapular internal/external rotation during bilateral arm elevation**. (A) Comparisons between the dominant and non-dominant scapulae; (B) Comparisons of the three groups of the dominant scapula; (C) Comparisons of the three groups of the non-dominant scapula. D: dominant; ND: non-dominant; painD:pain on dominant side; painND: pain on non-dominant side; painBoth: pain on both sides.

The patterns of scapular anterior/posterior tilt, upward/downward rotation, and internal/external rotation of the dominant and non-dominant arms in three groups of patients are illustrated in Figure 2, 3 and 4. In general, the scapula rotated upwards and externally and tilted posteriorly with increased arm elevation. The range of movement was 29° for the scapular upward/downward rotation, 13° for internal/external rotation and 12° for posterior/anterior tilt (Table 2). The results of three-way repeated measures ANOVA identified no significant three-way interaction for any of the scapular movements. However, scapular posterior tilt was significantly influenced by arm dominance (F = 14.29, P = 0.001) and by the interaction of arm dominance and elevation angle (F = 4.92, P = 0.002), while scapular upward/downward rotation was influenced by the interaction of arm dominance and elevation angle (F = 3.08, P = 0.02) (Table 3). The post-hoc analyses showed that non-dominant scapula tilted more posteriorly than the dominant scapula during arm elevation at 120°(mean difference [99% CI]: -6.04° [-11.92°,-0.15°]), and during arm lowering at 120° (mean difference [99% CI]: -5.51° [-10.98°,-0.05°]) (Figure 2). The location of neck pain did not show any significant influence on the scapular movement.

**Table 2:** Range of scapular movement during 30°-120° arm elevation in three groups of patients with chronic neck pain: mean (sd)

	Pain on dominant side (n = 9)	Pain on non-dominant side (n = 9)	Pain on both sides (n = 12)
Scapular posterior/anterior tilt			
Dominant scapula	5.13° (2.72°)	11.63° (6.25°)	7.44° (3.02°)
Non-dominant scapula	4.88° (2.68°)	7.12° (4.05°)	6.42° (2.84°)
Scapular upward/downward rotation			
Dominant scapula	25.78° (6.24°)	22.56° (10.21°)	28.03° (3.84°)
Non-dominant scapula	28.91° (8.35°)	25.13° (13.07°)	27.21° (3.66°)
Scapular internal/external rotation			
Dominant scapula	11.73° (4.31°)	9.00° (5.01°)	12.50° (5.48°)
Non-dominant scapula	12.82° (5.93°)	10.23° (6.05°)	11.89° (6.29°)

**Table 3:** Comparisons of scapular kinematics during bilateral arm elevation in subjects with chronic neck pain.^a^

Scapular kinematics	Effect	F	P	Observed power
Internal/external rotation	Dominance^b^	0.84	0.37	0.14
	Phase^b^	8.91	0.00*	1.00
	Group^b^	0.62	0.55	0.14
	Dominance*Group	0.40	0.68	0.11
	Phase*Group	1.37	0.21	0.69
	Dominance*Phase	2.35	0.00*	0.71
	Dominance*Phase*Group	0.61	0.86	0.31

Downward/upward rotation	Dominance^b^	0.25	0.62	0.08
	Phase^b^	36.80	0.00*	1.00
	Group^b^	0.70	0.50	0.16
	Dominance*Group	1.13	0.34	0.23
	Phase*Group	1.59	0.12	0.78
	Dominance*Phase	3.08	0.02*	0.84
	Dominance*Phase*Group	0.68	0.78	0.35

Posterior/anterior tilt	Dominance^b^	14.29	0.001*	0.95
	Phase^b^	18.68	0.00*	1.00
	Group^b^	1.70	0.20	0.33
	Dominance*Group	0.96	0.40	0.20
	Phase*Group	1.08	0.40	0.56
	Dominance*Phase	4.92	0.002*	0.97
	Dominance*Phase*Group	1.72	0.09	0.81

^a ^Repeated measures ANOVA

^b ^Dominance effect: right handed vs. left handed; Phase effect: humeral ascending and descending phases; Group effect: neck pain on dominant side/non-dominant side/both sides

*: P value < 0.05

### Electromyographic activities of the SCM and UT

**Figure 5:**
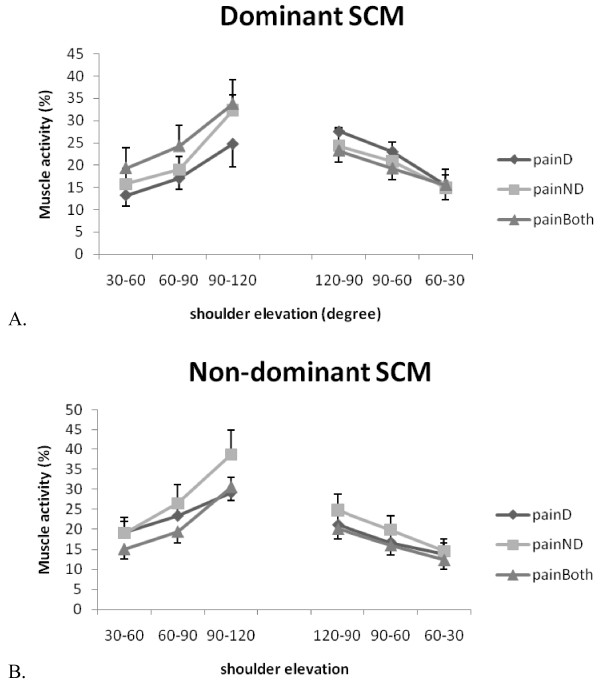
**Sternocleidomastoid muscle activities during bilateral arm elevation**. (A) Dominant side (B) Non-dominant side. SCM: sternocleidomastoid muscle; D: dominant; ND: non-dominant; painD:pain on dominant side; painND: pain on non-dominant side; painBoth: pain on both sides.

**Figure 6:**
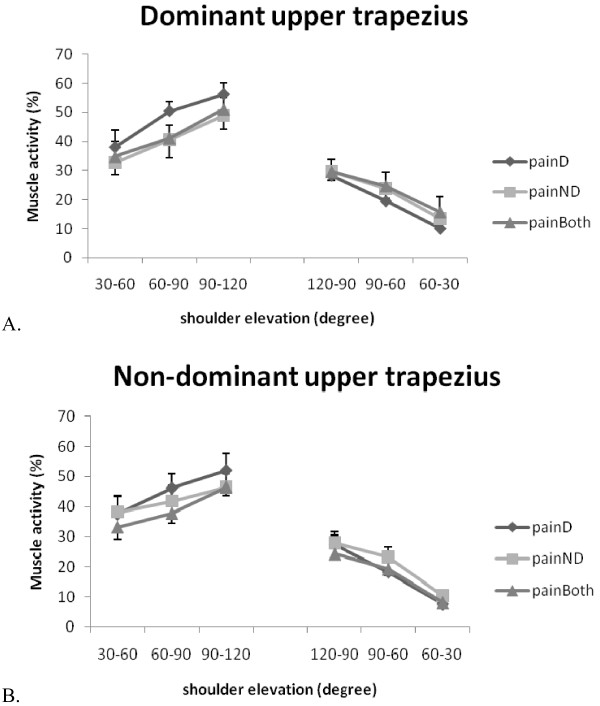
**Upper trapezius muscle activities during bilateral arm elevation**. (A) Dominant side (B) Non-dominant side. D: dominant; ND: non-dominant; painD:pain on dominant side; painND: pain on non-dominant side; painBoth: pain on both sides.

The EMG activities of the sternocleidomastoid and upper trapezius muscles increased with the arm ascending movement and vice versa (Table 4, Figure 5 and 6). However, three-way repeated measures ANOVA showed no effect of the neck pain location or the handedness on the activation of the sternocleidomastoid and upper trapezius muscles (Table 5).

**Table 4:** Sternocleidomastoid and upper trapezius muscle activities during bilateral arm elevation task: mean (sd)

Sternocleidomastoid muscle				
**Side**	**Humeral elevation angle (°)**	**Pain on dominant side (n = 9)**	**Pain on non-dominant side (n = 9)**	**Pain on both sides (n = 12)**

Dominant side	30-60	13.23 (7.33)	15.73 (10.64)	19.35 (15.77)
	60-90	17.1 (7.81)	18.98 (8.83)	24.26 (16.21)
	90-120	24.83 (15.42)	32.4 (10.19)	33.76 (18.79)
	120-90	27.62 (20.79)	24.35 (12.09)	23.28 (16.47)
	90-60	23.04 (19.00)	20.81 (12.94)	19.25 (15.22)
	60-30	15.62 (10.12)	14.87 (8.92)	15.55 (12.05)

Non-dominant side	30-60	19.11 (8.40)	19.09 (11.41)	15.07 (8.36)
	60-90	23.36 (8.88)	26.49 (14.31)	19.44 (9.55)
	90-120	29.21 (10.97)	38.68 (19.07)	30.62 (12.03)
	120-90	21.09 (9.55)	24.79 (12.16)	20.13 (8.81)
	90-60	16.67 (9.12)	19.87 (10.62)	16.03 (8.74)
	60-30	13.87 (7.91)	14.54 (9.07)	12.33 (8.27)

Upper trapezius muscle				

Side	Humeral elevation angle (°)	Pain on dominant side (n = 9)	Pain on non-dominant side (n = 9)	Pain on both sides (n = 12)

Dominant side	30-60	38.05 (19.72)	32.81 (12.94)	34.74 (14.53)
	60-90	50.50 (10.86)	40.51 (17.85)	41.14 (14.09)
	90-120	56.33 (11.27)	48.99 (14.37)	51.06 (14.59)
	120-90	28.26 (7.24)	29.76 (9.56)	29.55 (8.36)
	90-60	19.46 (10.69)	23.87 (10.27)	24.64 (8.08)
	60-30	10.02 (9.01)	13.42 (6.86)	15.58 (7.66)

Non-dominant side	30-60	37.44 (17.86)	38.24 (12.05)	33.17 (10.59)
	60-90	46.10 (8.14)	41.87 (16.88)	37.76 (10.63)
	90-120	52.03 (7.97)	46.54 (13.66)	46.47 (8.68)
	120-90	27.33 (8.74)	28.08 (9.98)	24.28 (6.62)
	90-60	18.30 (10.81)	23.29 (9.94)	19.24 (7.91)
	60-30	7.65 (6.99)	10.32 (8.23)	8.19 (5.79)

**Table 5:** Comparisons of EMG muscle activity during bilateral arm elevation in subjects with chronic neck pain.^a^

Muscle	Effect	F	P	Observed power
Sternocleidomastoid muscle	Dominance^b^	0.01	0.95	0.50
	Phase^b^	22.57	0.00*	1.00
	Group^b^	0.21	0.81	0.08
	Dominance*Group	0.42	0.66	0.11
	Phase*Group	1.07	0.41	0.49
	Dominance*Phase	1.24	0.32	0.36
	Dominance*Phase*Group	1.06	0.41	0.48

Upper trapezius muscle	Dominance^b^	0.01	0.95	0.05
	Phase^b^	129.17	0.00*	1.00
	Group^b^	1.07	0.34	0.22
	Dominance*Group	0.24	0.79	0.08
	Phase*Group	1.67	0.12	0.72
	Dominance*Phase	0.72	0.62	0.21
	Dominance*Phase*Group	0.59	0.82	0.26

^a ^Repeated measures ANOVA

^b ^Dominance effect: right handed vs. left handed; Phase effect: humeral ascending and descending phases; Group effect: neck pain on dominant side/non-dominant side/both sides

*: P value < 0.05

## Discussion

To our knowledge, this is the first study examining the effect of neck pain location and hand dominance on kinematics and muscle activities of the neck and shoulder area in patients with chronic neck pain. We hypothesized that movement of the scapula and the activities of the sternocleidomastoid and upper trapezius muscles would differ between dominant and non-dominant arm, and between individuals with different location of neck pain (on dominant, non-dominant, or on both sides). The results of our study supported the first hypothesis that scapular posterior/anterior tilt and downward/upward rotation were affected by the side-dominance. However, individuals with different location of neck pain showed a similar pattern of scapular movement and muscle activation pattern. We recognized that our sample size was small for the comparisons between three different groups and between two arms, and that the range of scapular movement we dealt with was small but with large between-subject variations (large standard deviations as shown in Figure 2, 3 and 4). However, we believed our measurement was appropriate with a measurement accuracy of 0.3° to 0.7° and a standard error of measurement between 0.56° and 4.22°.

Movement of the scapula differ between patients with chronic neck pain and healthy controls [1,3]. However, most of the studies assessed the kinematic performance without considering the effect of neck pain location and hand dominance. In healthy subjects, Crosbie et al. (2008) identified greater upward rotation movement of the non-dominant scapula during the unilateral arm elevation task [7], and suggested that side dominance should not be disregarded when evaluating the shoulder kinematics. Matsuki et al. (2011) identified as much as 10° more scapular downward rotation at rest and 4° more upward rotation in dominant shoulders during arm elevation [8]. These authors suggested that these normal variations in bilateral scapular movement could not be overlooked in clinical assessment [8]. In the current study, the effect of hand dominance was evident for the scapular posterior tilt, and the interaction of hand dominance and arm elevation angle significantly influenced the movement pattern of the scapular posterior/anterior tilt and upward/downward rotation. In patients with chronic neck pain, the non-dominant scapula tilted more posteriorly during arm ascending (120°) and descending (120°). Compared with the dominant scapula, the non-dominant scapula showed slightly larger downward rotation (5.2° vs. 3.7°) at the end of the lowering phase (60° to 30°). Our results agreed with Crosbie et al. and Matsuki et al. that dominant and non- dominant scapula moved differently during arm elevation, and that scapular rotation in the frontal and sagittal planes seemed to increase in the non-dominant arm. Since we did not find any effect of neck pain location on the scapular kinematics, these bilateral differences could be normal variations in both contractile and soft tissues [7,8]. The asymmetry of the scapular kinematics should be considered when assessing patients with chronic neck pain.

Despite the significant impact of hand dominance on scapular movement, we did not identify any association between neck pain location and scapular kinematics. The average NDI score of the subjects in this study was 13.84. According to Vernon's study, an NDI score between 5 and 14 was in the category of mild disability [24]. The subclinical pain presented by our participants might partly account for the non-significant findings regarding the effect of neck pain location. A small sample size of 9 to 12 subjects in each group did not provide sufficient statistical power for the comparisons we tried to examine. A larger sample size and subjects with higher neck pain scores might be needed to explore this issue further. In addition, the measurement repeatability ICC value less than 0.6 for some of the outcome variables might contribute to the non-significant findings.

The amplitude of SCM and UT muscle activities increased with arm elevation, and this pattern of muscle activation was consistent with previous data [9]. Despite previous data demonstrating differences in EMG amplitude of the left and right scapular muscles [10], and in trapezius, serratus anterior, and middle deltoid muscle activities for dominant and non-dominant arm [9], our study failed to identify the effect of handedness or location of neck pain on scapular muscle activities in subjects with chronic neck pain. A small sample size of subjects might account for the insignificant findings on muscle activation patterns.

In this study, thirty subjects who experienced posture-related, chronic neck pain were recruited, and hand dominance was found to significantly affect the movement of the scapula. We therefore suggested that handedness should be appreciated when evaluating the movement performance of the shoulder complex in this patient population. Because of the small sample size and the specific pain characteristics of our subjects, generalization of our findings should be cautious. In addition, the small sample size of each group compromised our statistical power (Table 3 and 5). These factors should be taken into consideration in future research.

## Conclusions

Hand dominance could have an influence on the scapular kinematics, which should be considered when describing and comparing neuromuscular characteristics in individuals with chronic neck pain.

## List of abbreviations

EMG: electromyography; NDI: Neck Disability Index; SD: standard deviation; SCM: sternocleidomastoid; UT: upper trapezius; ANOVA: analysis of variance; ICC: intraclass correlation coefficient; RMS: root-mean square; 95% CI: 95% confidence interval.

## Competing interests

The authors confirm that there are no known conflicts of interest associated with this publication and there has been no significant financial support for this work that could have influenced its outcome.

## Authors' contributions

YFS and YHK designed the study. YHK performed data acquisition. YHK and YFS analyzed and interpreted the data. YFS and YHK wrote the manuscript. YHK and YFS read and approved the manuscript.

## Pre-publication history

The pre-publication history for this paper can be accessed here:

http://www.biomedcentral.com/1471-2474/12/267/prepub
